# Advances in the study of key cells and signaling pathways in renal fibrosis and the interventional role of Chinese medicines

**DOI:** 10.3389/fphar.2024.1403227

**Published:** 2024-12-02

**Authors:** Lijuan Liang, Youjun Mi, Shihan Zhou, Aojian Yang, Chaoyu Wei, Enlai Dai

**Affiliations:** ^1^ Gansu University of Chinese Medicine, Lanzhou, China; ^2^ Key Laboratory of Dunhuang Medicine and Translation, Ministry of Education, Lanzhou, China; ^3^ Institute of pathophysiology, School of Basic Medical Sciences, Lanzhou University, Lanzhou, China

**Keywords:** renal fibrosis, chronic kidney disease, pathogenesis, signaling pathways, traditional Chinese medicine, treatment

## Abstract

Renal fibrosis (RF) is a pathological process characterized by the excessive accumulation of extracellular matrix (ECM), which triggers a repair cascade in response to stimuli and pathogenic factors, leading to the activation of molecular signaling pathways involved in fibrosis. This article discusses the key cells, molecules, and signaling pathways implicated in the pathogenesis of RF, with a particular focus on tubular epithelial cells (TECs), cellular senescence, ferroptosis, autophagy, epithelial-mesenchymal transition (EMT), and transforming growth factor-β(TGF-β)/Smad signaling. These factors drive the core and regulatory pathways that significantly influence RF. A comprehensive understanding of their roles is essential. Through a literature review, we explore recent advancements in traditional Chinese medicine (TCM) aimed at reducing RF and inhibiting chronic kidney disease (CKD). We summarize, analyze, and elaborate on the important role of Chinese herbs in RF, aiming to provide new directions for their application in prevention and treatment, as well as scientific guidance for clinical practices.

## 1 Introduction

Globally, the societal burden of CKD is increasing every year. Between 1990 and 2017, the prevalence across all ages increased by 29%, while mortality for all ages rose by 41.5%, suggesting that CKD is becoming a global public health problem (Collaboration et al., 2020). China accounts for nearly one-fifth of these patients, and RF is a common pathological process in the progression of CKD and end-stage renal disease (ESRD) (Webster et al., 2017). RF encompasses a series of pathological processes, including tubular epithelial cell (TEC); podocyte, pericyte, and endothelial cell injury; fibroblast activation and collagen deposition; glomerulosclerosis; tubular atrophy; and capillary rarefaction (Wang et al., 2018a). These pathological changes lead to the progressive deterioration of renal function, ultimately resulting in ESRD.

RF occurs in response to various stimuli, including trauma, infection, mechanical obstruction, exogenous organisms, toxins, and genetic disorders. These stimuli trigger a repair cascade response, leading to inflammation, activation of myofibroblasts, and ECM deposition (Cellular senescence and the senescence, 2022). Hypertension and diabetes are the two main causes of RF. However, treatment options for RF are limited and often expensive, placing a heavy financial burden on patients and healthcare systems. Despite the availability of several therapies, outcomes remain poor. Therefore, in this article, we reviewed the pathogenesis of RF and recent intervention studies using Chinese herbs to treat this condition. Our aim is to highlight the significant advantages that Chinese herbs may offer in blocking or reversing the progression of RF.

## 2 Progress in the study of RF

### 2.1 Cellular senescence and senescence-related secretory phenotype (SASP)

**FIGURE 1:**
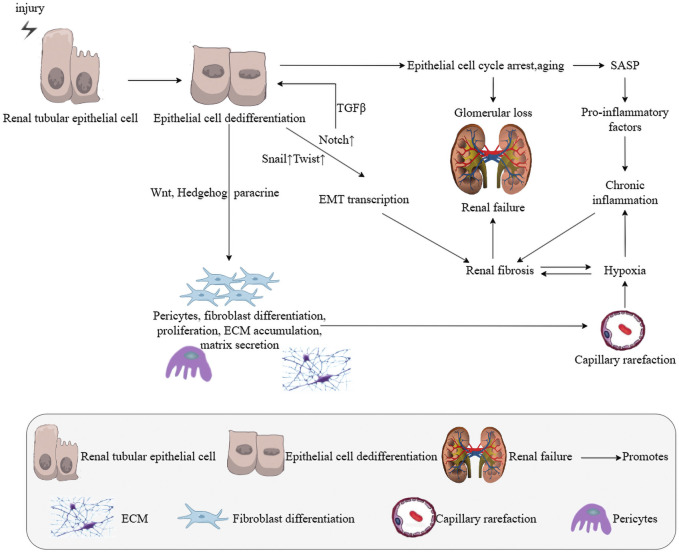
Key cells and signaling pathways activated in renal fibrosis.

The mechanisms of RF involve all kidney cells, numerous signaling pathways, and complex cellular interactions. Recently, the roles of cellular senescence and the SASP in progressive RF have become increasingly evident (Cellular senescence and the senescence, 2022). Cellular senescence is the irreversible exit of cells from the cell cycle into a stable state of growth arrest, characterized by changes in cell cycle proteins, increased expression of senescence-associated β-galactosidase (SA-β-gal), and double-stranded DNA breaks. Transiently generated senescent cells limit overproliferation and secrete specific factors to enhance cellular repair. However, incomplete recovery results in excessive accumulation of senescent cells, leading to slowed tissue regeneration and increased secretion of various inflammatory and profibrotic mediators, a condition known as the SASP (Docherty et al., 2019). The mechanisms of RF include oxidative stress, cellular senescence, epigenetic disorders, chronic inflammation, autophagy, impaired energy metabolism, and apoptosis. Among these, cellular senescence—especially TEC senescence—is a major cause of RF progression (Figure 1). RF pathology is characterized by glomerulosclerosis, tubular atrophy, interstitial fibrosis, capillary rarefaction, and a decrease in the number of renal units. When senescent cells are removed, nuclear factor κB (NF-κB) levels decrease, inflammatory cells infiltrate, and TGF-β/Smad signaling and EMT are activated. This suggests that anti-aging therapy may be a potential target for treating RF.

Senescent cells promote oncogenesis in neighboring precancerous cells by secreting inflammation-associated and oncogene-associated factors. Sufficient DNA damage leads to the formation of the SASP (Cellular senescence and the senescence, 2022). SASP production is amplified through a cascade of NF-κB activation and interleukin-1α (IL-1α) production. Consequently, SASP factors accumulate through this cascade, creating a persistent chronic inflammatory state during aging (Cellular senescence and the senescence, 2022). Chronic inflammation, in turn, ultimately contributes to fibrosis.

SASP factors are categorized into four main groups: proteases, chemokines, cytokines, and growth factors (Cellular senescence and the senescence, 2022). The composition of SASP factors varies depending on different stressors, cell types, and disease stages. According to the theory of antagonistic pleiotropy, during the early stages of cellular senescence, the SASP recruits immune cells, eliminates damaged cells, restores the normal cell cycle, and promotes tissue repair. However, if cell repair fails, senescent cells continue to accumulate and secrete large amounts of SASP factors through positive feedback loops. This persistent inflammatory state promotes fibrosis (Wang et al., 2018b). In RF, during EMT, TECs are damaged and secrete inflammatory factors locally. This secretion induces macrophage recruitment and fibroblast activation, leading to the production of matrix metalloproteinases (MMPs), especially MMP-9. MMP-9 causes basement membrane damage and local degradation of laminin and type IV collagen, promoting the dedifferentiation of TECs (Chang et al., 2012). Concurrently, growth factors such as TGF-β, platelet-derived growth factor (PDGF), epidermal growth factor (EGF), and fibroblast growth factor 2(FGF2) are released to remove deleterious factors and facilitate tissue repair. Blocking the expression of the TGF-β signaling pathway and MMP-9, as well as inhibiting EMT, can be therapeutic strategies (Yan et al., 2021). Additionally, the secretion of TGF-β1 and MMP-9 by the SASP has been shown to induce endothelial-mesenchymal transition and fibrosis markers in primary mouse renal peritubular endothelial cells. Inhibition of EMT and reduction of SASP expression suggest that the SASP is a crucial mediator between cellular senescence and EMT (Cellular senescence and the senescence, 2022). Cellular senescence and SASP factors play key roles in the development of RF.

### 2.2 Renal tubular epithelial cells (RTECs)

RTECs, the primary resident cells of the kidney, are highly susceptible to injuries, including hypoxia, proteinuria, and toxins (Huang et al., 2023). Traditionally, RTECs were considered mere victims of RF. RTECs play a crucial role in responding to injury, with damaged RTECs serving as key contributors to the progression of fibrotic kidney disease. Severe injury leads to extensive apoptosis in RTECs, while some surviving cells undergo transformations, become fibrogenic, and contribute to interstitial inflammation and fibrosis by secreting profibrogenic growth factors, such as TGF-β1 and connective tissue growth factor (CTGF). RTECs can maintain homeostasis at the DNA, protein, and organelle levels in response to both exogenous and endogenous insults (Guo et al., 2024).

RTECs play a crucial role in RF. Under adverse conditions such as injury, TECs change to cope with the damage by transforming into a secretory phenotype. This leads to the release of various bioactive molecules that promote the recruitment of inflammatory cells, activation of fibroblasts, and loss of endothelial cells, ultimately driving tubulointerstitial inflammation and fibrosis. Research has shown that selective injury to the renal tubules promotes fibrosis, inflammation, and capillary rarefaction, making it a key link between acute kidney injury (AKI) and CKD (Huang et al., 2023; Petreski et al., 2023). Additionally, abnormal repair of TECs after injury results in the acquisition of a fibrotic phenotype by these cells.

Meanwhile, during injury, the RTEC innate immune system further exacerbates the immune response. Necroinflammation forms a self-amplifying loop between tubular cell death and interstitial inflammation, leading to the worsening of kidney injury. TECs transition from being victims of injury to drivers of the progression from AKI to CKD. Additionally, damaged tubular cells directly contribute to interstitial inflammation and fibrosis through new mechanisms related to partial EMT, cell cycle arrest, and metabolic disorders (Liu et al., 2018).

Hypoxic RTECs can promote fibrosis through various mediators, including HIF-1α (Li et al., 2024). The Ras oncogene inhibitor, RAS protein activator like-1 (RASAL1), can alleviate interstitial fibrosis by methylating DNA methyltransferase 1 (DNMT1) (Huang et al., 2023). Another chromatin regulator, histone deacetylase, can regulate the pro-inflammatory functions and fibrotic processes in tubulointerstitial injury (Shen and Zhuang, 2022). Therefore, TECs are considered key inflammatory and pathogenic cells driving the progression of RF. Understanding the mechanisms by which TEC injury leads to inflammation and fibrosis may offer new therapeutic approaches to halt the progression of CKD.

### 2.3 Ferroptosis and RF

Ferroptosis is a form of regulated cell death that is distinct from apoptosis and has been shown to play a significant role in AKI and tumors (Belavgeni et al., 2020). A reciprocal relationship exists between ferroptosis and RF, wherein each can promote and convert into the other. In the renal tissue of CKD, lipid peroxidation, necroinflammatory responses, and iron overload lead to the destruction of parenchymal cells, resulting in pathological fibrosis and accelerating the progression of RF. Thus, oxidative stress, inflammation, and iron overload are critical links between ferroptosis and RF. Proximal tubular (PT) cells, when exposed to adverse factors, can develop a unique pro-inflammatory state that predisposes them to ferroptosis, such that even mild stimuli may trigger this process, impairing the normal repair mechanisms of renal cells and ultimately inducing RF (Guo et al., 2024). Ferroptosis occurs during the progression of RF and plays a pivotal role in facilitating fibroblast differentiation. Ferroptosis takes place throughout the development of RF and contributes significantly to the disease’s progression (Zhang et al., 2021).

Ide et al. (2021) found that iron overload in severely injured proximal tubular (PT) cells can trigger ferroptosis, leading to a significant accumulation of inflammatory PT cells. Sustained inflammation is a foundational element of organ fibrosis and contributes to RF, thereby exacerbating kidney damage, indicating a close relationship between RF, inflammation, and renal injury. These findings suggest that enhancing renal repair and regeneration by modulating the ferroptotic stress response pathways may prevent the progression to CKD and RF. Ferroptosis is closely linked to the occurrence, progression, and prognosis of CKD and can be targeted as a therapeutic strategy for RF. Furthermore, the research indicates that ferroptosis can promote irreversible changes in cellular states, leading to pathological accumulation and contributing to the progression of RF through non-lethal pathways, in addition to inducing a malignant feedback loop of necroinflammatory responses. This process, beginning as an initiator of fibrotic events and later becoming a key coordinator of the fate of regenerative and repair cells, is likely to occur similarly in the kidneys and other organs and tissues.

### 2.4 Autophagy in RF

The term “autophagy,” first proposed by Christian de Duve in 1963, is derived from the Greek term meaning “self-eating” (Rabinovich-Nikitin et al., 2023). Autophagy is a highly conserved lysosomal pathway responsible for the degradation of cytoplasmic components that can be triggered by metabolic stress, genotoxicity, or hypoxia and functions as a critical adaptive mechanism for cell survival (Li et al., 2021a). Autophagy is associated with various diseases, including neurodegenerative diseases, cancer, inflammation, autoimmune disorders, and RF (Mizushima and Levine, 2020; Dong et al., 2021; Yamazaki et al., 2021; Yamamoto et al., 2021).

Autophagy is a highly conserved lysosomal degradation pathway that plays a crucial role in maintaining cellular homeostasis in all major types of renal cells, including tubular cells, podocytes, mesangial cells, and endothelial cells in the glomerulus (Zhao et al., 2019). Recent evidence suggests that dysregulation of autophagy may contribute to the pathogenesis of RF and related kidney diseases.

The activation of autophagy provides a protective effect on renal cells under stress conditions (Tan et al., 2018). In the absence of sufficient autophagy, the kidney becomes more vulnerable to damage, leading to impaired renal function, the accumulation of damaged mitochondria, premature kidney aging, and the progression of RF (Gui et al., 2021). However, other studies have demonstrated that prolonged autophagy activation following severe injury can harm the kidney, inducing renal cell senescence and promoting RF through the secretion of profibrotic cytokines (Zheng et al., 2019).

The activation of autophagy exerts a protective effect on renal cells under stress conditions (Tan et al., 2018). When autophagy is deficient, the kidney becomes more susceptible to damage, resulting in impaired renal function, accumulation of damaged mitochondria, premature aging of the kidney, and exacerbation of RF (Gui et al., 2021). However, other studies have shown that prolonged activation of autophagy following severe injury can be detrimental to the kidney, leading to renal cell senescence and promoting RF through the secretion of profibrotic cytokines (Zheng et al., 2019).

As a key stress-responsive system, autophagy has been implicated in the pathogenesis of various kidney diseases, including RF (Choi, 2020). Although autophagy is not essential for kidney development (Suman et al., 2024), it is crucial for the function of resident cells in the adult kidney and is closely associated with the progression of RF (Tang et al., 2020).

Unilateral ureteral obstruction (UUO) is a widely recognized model for investigating RF. Evidence suggests that autophagy is activated in the renal tubules of UUO mice, accompanied by tubular cell apoptosis (Li et al., 2010; Livingston et al., 2016; Jung et al., 2022). In this context, autophagy and apoptosis synergistically contribute to tubular atrophy and nephron loss. Oxidative stress-induced mitochondrial damage may stimulate both autophagy and apoptosis in renal tubules, potentially contributing to tubular degradation during the progression of UUO (Jung et al., 2022). Koesters et al. (2010), using a tetracycline-controlled mouse model with specific overexpression of TGF-β1 in renal tubules, demonstrated that sustained TGF-β1 expression promoted autophagy in the tubules, leading to tubular dedifferentiation and extensive peritubular fibrosis. Notably, these degenerating cells were TUNEL-negative for apoptosis, suggesting that autophagy may be a key driver of tubular atrophy in TGF-β1-induced RF (Koesters et al., 2010). Through pharmacological and genetic inhibition strategies, we further confirmed the profibrotic role of autophagy in both the UUO mouse model and TGF-β1-treated primary PTECs (Livingston et al., 2016). Autophagy remained persistently activated in proximal tubules following UUO. Pharmacological and genetic inhibition of autophagy attenuated interstitial fibrosis, tubular apoptosis, macrophage infiltration, and FGF2 production. In primary PTEC cultures, TGF-β1 induced fibronectin accumulation and cell death in an autophagy-dependent manner (Livingston et al., 2016). A recent study by Yan et al. further elucidated the link between sustained activation of autophagy in tubular epithelial cells and lipid accumulation during RF (Yan et al., 2018). Autophagy inhibitors significantly reduced UUO-induced lipid accumulation in tubular cells, thereby alleviating interstitial fibrosis, tubular apoptosis, and tubular dedifferentiation. This fibrosis-associated lipid accumulation was found to be unrelated to the lipophagic lysosomal pathway but dependent on BECN1. These findings underscore the pivotal role of autophagy in regulating lipid metabolism in renal tubular cells. Additionally, autophagy’s involvement in fibroblast activation and its profibrotic effects were confirmed in the UUO kidney and TGF-β1-treated cultured renal fibroblasts. Protein kinase C (PKC)-α drives renal fibroblast activation and RF by stimulating autophagic flux (Bao et al., 2024). Targeting autophagy as a therapeutic approach for CKD, particularly focusing on the roles of TGF-β1 in autophagy and kidney fibrosis, holds promise for future interventions.

### 2.5 Hypoxia and RF

Hypoxia, a common pathological hallmark of advanced kidney disease, plays a key role in the development of RF. Chronic hypoxia in renal disease is driven by mechanisms such as microvascular rarefaction, decreased oxygen supply due to glomerular injury, an imbalance of vasoactive substances, and increased oxygen consumption. These mechanisms ultimately lead to chronic hypoxia (Wang et al., 2022a). TECs are particularly susceptible to hypoxia due to their high metabolism and increased oxygen demand (Natarajan et al., 2021). RF and hypoxia influence and interact with each other: hypoxia initiates an inflammatory response that increases ECM proteins and promotes EMT, which contributes to RF (Figure 1). Conversely, excessive deposition of ECM in RF exacerbates hypoxia, creating a vicious cycle that leads to tissue destruction. Several factors and pathways are involved in this process, including microRNAs, hypoxia-inducible factors (HIFs), PI3K/Akt, PKC/ERK, TGF-β, Notch, NF-κB, angiotensin II/reactive oxygen species (Ang II/ROS), adenosine (ADO), IL-6, IL-18, and KIM-1 (Wang et al., 2022a; Hu et al., 2021). Hypoxia, along with these signaling pathways and their interactions, promotes fibroblast activation, proliferation, EMT, and ECM deposition during scarring, ultimately culminating in RF. Intervening in hypoxia can help delay or halt RF and improve clinical outcomes in CKD patients (Liu et al., 2017).

HIF is a key regulator in hypoxic environments and plays a crucial role in the pathophysiology of RF (Liu et al., 2022a). HIF regulates various target genes, including microRNAs, which are involved in renal injury and repair. It triggers EMT and induces the expression of fibrogenic genes. Additionally, the hypoxic and fibrotic microenvironment can disrupt cell signaling and lead to epigenetic modifications that promote fibroblast proliferation, activation, and fibroblast-to-myofibroblast transformation (Huang et al., 2023). Experimental studies have found that HIF-1 may drive RF by upregulating the expression of lysyl oxidases (LOXs) (Higgins et al., 2007). HIF-1-mediated LOXs increase the expression of ECM-modifying factors, promote Snail1 activation and EMT, and act as important mediators in the profibrotic HIF signaling pathway in renal epithelial cells (Liu et al., 2017).

In RF, the ECM significantly increases. Myofibroblasts, which are the primary producers of ECM, originate mainly from pericytes that stabilize capillaries. During fibrosis, pericytes are activated and contribute to the worsening of hypoxia. Therefore, targeting pericytes to correct hypoxia and mitigate fibrosis represents a novel approach to the treatment of RF (Liu, 2011).

### 2.6 Myofibroblasts

Myofibroblasts are a group of cells located in the renal interstitium with both fibroblast and smooth muscle cell characteristics. They are the main effector cells for ECM production, as well as the main cells in renal interstitial fibrosis (RIF). Upon activation, myofibroblasts express alpha-smooth muscle actin (α-SMA), which secretes large amounts of ECM and promotes tissue repair (Yuan et al., 2019). The continuous activation of myofibroblasts leads to ECM deposition and exacerbates RF. Myofibroblasts are absent in normal kidneys but are detected in large numbers in RF (Arai and Yanagita, 2020). Profibrotic cytokines in the fibrotic microenvironment convert many types of cells in the kidney to myofibroblasts and promote their proliferation (Qian and Youhua, 2018). It has been found that approximately 50% of fibroblasts can be converted to myofibroblasts when induced by hypoxia, inflammatory cytokines, and TGF (Duan et al., 2020). During RF, many mediators are involved in fibroblast–myofibroblast transformation (FMT). It has been reported that TGF-β1-induced upregulation of SRY-box transcription factor 9 (Sox9) leads to the proliferation and activation of renal fibroblasts, resulting in RF, implying that Sox9 may be an innovative target for the prevention of RF (Docherty et al., 2019). Furthermore, sphingosine kinase 2 synergizes with Fyn by targeting TGF-β1-stimulated normal rat renal interstitial fibroblasts (NRK-49 F) (Duan et al., 2020) in the transcription activator 3 and protein kinase B (Akt) signaling pathways that drive fibroblast activation. In addition, the lysophosphatidic acid signaling pathway has been reported to promote renal fibroblast proliferation and myofibroblast differentiation via the CTGF signaling pathway in UUO mice (Hirata et al., 2021). Epidermal growth factor receptor 2 (EGFR2) promotes renal fibroblast proliferation and activation through the CTGF signaling pathway *in vitro* (Li et al., 2019). Increasing evidence suggests that multiple cell types are involved in the origin of renal myofibroblasts and many mediators and involved in renal myofibroblast activation during renal fibrogenesis, suggesting that pharmacologically targeted interventions against renal myofibroblast origin and activation would be helpful in the treatment of RF.

Myofibroblasts are cells located in the renal interstitium that possess characteristics of both fibroblasts and smooth muscle cells. They are the primary effector cells responsible for ECM production and play a central role in RF. Upon activation, myofibroblasts express α-SMA and secrete large amounts of ECM, which promotes tissue repair (Yuan et al., 2019). However, persistent activation of myofibroblasts leads to excessive ECM deposition and exacerbates RF. While myofibroblasts are absent in normal kidneys, they are abundantly present in RF. Profibrotic cytokines in the fibrotic microenvironment convert various kidney cell types into myofibroblasts and promote their proliferation (Qian and Youhua, 2018)]. Studies have shown that approximately 50% of fibroblasts can transform into myofibroblasts when exposed to hypoxia, inflammatory cytokines, and TGF (Duan et al., 2020). During RF, several mediators are involved in FMT. For instance, TGF-β1-induced upregulation of Sox9 has been linked to the proliferation and activation of renal fibroblasts, suggesting Sox9 as a potential target for RF prevention. Additionally, sphingosine kinase 2, in synergy with Fyn, targets TGF-β1-stimulated normal rat renal interstitial fibroblasts (NRK-49F) through the transcription activator 3 and protein kinase B (Akt) signaling pathways, driving fibroblast activation (Duan et al., 2020). Furthermore, the lysophosphatidic acid signaling pathway has been shown to induce renal fibroblast proliferation and myofibroblast differentiation through the CTGF signaling pathway in UUO mice (Hirata et al., 2021). EGFR2 also promotes renal fibroblast proliferation and activation through the CTGF signaling pathway *in vitro* (Li et al., 2019).

### 2.7 EMT

The EMT is the biological process through which TECs acquire a mesenchymal phenotype, which is a hallmark of RF (Sheng and Zhuang, 2020). The EMT plays a crucial role in organ fibrosis, tissue regeneration, wound healing, and cancer progression. Increasing evidence suggests that epithelial cells significantly contribute to RF through EMT. During EMT, TECs lose their intercellular tight junctions, normal morphology, and epithelial cell markers such as E-cadherin and instead express mesenchymal markers like α-SMA and vimentin (Hadpech and Thongboonkerd, 2024). TGF-β1 is a key inducer of the EMT and RF (Figure 1) (Liu et al., 2021).

RF is characterized by the EMT of TECs. During RF, TECs undergo part of the EMT program, expressing markers associated with both epithelial and mesenchymal cells. The EMT in fibrosis leads to a blockage of the G2 phase of the TEC cell cycle and a reduction in transport proteins. Research has shown that the expression of Snail, a transcriptional regulator that induces the EMT, prolongs the G2 block in TECs, promotes mesenchymal expression, and leads to significant collagen deposition (Qi et al., 2021). Moreover, in a mouse model of RF, deletion of Twist1 or Snail1 in TECs inhibits the EMT, preserves TEC integrity, restores proliferation, and enhances repair and regeneration of the renal parenchyma, thereby attenuating mesenchymal fibrosis (Hu et al., 2022). These findings suggest that preventing fibrosis by inhibiting the EMT in TECs could be a viable strategy for managing chronic kidney injury.

Various forms of RF are marked by differing degrees of TEC injury. This injury causes TECs to release growth factors, cytokines, chemokines, and MMPs, which initiate the host’s regenerative and reparative responses. These responses lead to vasodilation, basement membrane remodeling, and an increase in macrophages (Li et al., 2022a). Damaged TECs undergoing apoptosis and stress trigger the EMT. However, the EMT exacerbates TEC damage, impairs their function, and induces cell cycle arrest. This process further amplifies the host damage response, immune response, and recruitment of myofibroblasts, creating a vicious cycle of injury and host response that ultimately results in RF. Preventing the EMT in injured TECs can restore tubular epithelial function, reduce cell cycle arrest, and improve organ function, leading to a decrease in myofibroblasts and a reduction in ECM deposition (Sheng and Zhuang, 2020).

CKD is characterized by TEC injury, which results in the release of cytokines, TGF, and MMPs. This response leads to vasodilation, basement membrane remodeling, and increased macrophage infiltration (Li et al., 2022a). Damaged TECs, under stress and apoptosis, initiate the EMT. The EMT, in turn, exacerbates TEC damage, creating a vicious cycle that amplifies injury, immune responses, and the recruitment of myofibroblasts, ultimately resulting in RF (Zhou and Liu, 2016).

### 2.8 TGF-β/Smad signaling pathway in RF

RF is a major pathological feature of CKD. TGF-β1 is a central mediator of RF, and increasing research focuses on inhibiting TGF-β1 and its downstream target genes as a strategy for treating CKD (Chen et al., 2018a). The Smad-dependent signaling pathway is also crucial in the pathogenesis of CKD (Qian et al., 2022).

TGF-β/Smad signaling mediates inflammation and RF. TGF-β1 drives progressive RF by stimulating ECM production and inhibiting ECM degradation (Chen et al., 2018a). Myofibroblasts, which are the main source of ECM production, are activated as a key step in RF. TGF-β1 promotes RF by inducing the differentiation of quiescent fibroblasts into matrix-secreting myofibroblasts (Sun et al., 2016). Smad2 and Smad3 are principal downstream mediators of TGF-β1. Research indicates that Smad3 is primarily responsible for mediating RF, and its inhibition can prevent the onset of fibrosis (Chen et al., 2018a). Downregulation of Smad2/3 phosphorylation and upregulation of Smad7 can restore podocyte function and prevent RF by inhibiting TGF-β1 expression (Zhao et al., 2024). Smad4 is a key regulator of TGF-β1-mediated fibrosis and inflammation, interacting with Smad3 and Smad7 to influence their transcriptional activity in renal inflammation and fibrosis (Chen et al., 2018a). However, TGF-β1 is not the only mediator that activates Smad signaling in CKD (Qian et al., 2022). The TGF-β/Smad pathway is regulated by ubiquitination, where components of the ubiquitin–proteasome system can tightly control TGF-β/Smad signaling and RF by modifying specific ubiquitin-modifying enzymes (Zhao et al., 2023).

Smad2 and Smad4 also play important roles in RF. Studies have shown that knockdown of Smad2 and Smad4 can prevent the onset of RF. Specifically, deletion of Smad2 significantly reduces RF, tubular EMT, and myofibroblast marker levels, while decreasing the expression of Smad3 and TGF-β1 in mice with diabetic nephropathy (DN) (Loeffler et al., 2018). Additionally, Smad2 gene deletion promotes fibrosis by enhancing TGF-β/Smad3 signaling and increasing TGF-β1 auto-induction. On the other hand, deletion of Smad4 inhibits RF and reduces TGF-β1-induced collagen I expression (Meng et al., 2012). TGF-β signaling involves both canonical and non-canonical pathways, significantly influencing the progression of RF.

### 2.9 Bone morphogenetic protein-7 (BMP-7), Wnt/β-catenin pathway

BMP-7, a member of the TGF-β superfamily, is predominantly expressed in podocytes and TECs of the kidney (Wen et al., 2019). BMP-7 signaling includes both the Smad-mediated classical pathway and non-Smad pathways. Hyperglycemia has been shown to decrease both mRNA and protein levels of BMP-7 in TECs, with a negative correlation to RF (Liu et al., 2021). During RF, the expressions of BMP-7 and its receptor are reduced (Ji et al., 2020). Experimental studies have demonstrated that BMP-7 administration inhibits the phosphorylation of associated mitogen-activated protein kinase pathways and effectively mitigates renal injury in rat models of ureteral obstruction. Additionally, BMP-7 administration has been shown to alleviate RF in these models. In rats with single-dose streptozotocin-induced DN, BMP-7 restored the glomerular filtration rate (GFR), reduced proteinuria, and normalized histology, significantly reversing interstitial fibrosis (Ji et al., 2020). Therefore, BMP-7 is considered a negative regulator of organ fibrosis and a potential renoprotective factor.

TGF-β exerts profibrotic effects through interactions with other signaling pathways, including BMP-7, Wnt/β-catenin, and MAP kinases. Wnt proteins, a group of lipid-modified glycoproteins, play a crucial role in these pathways. Activation of the Wnt/β-catenin pathway, with β-catenin proteins acting as key mediators, is a hallmark of RF development. In UUO, the expression of 19 Wnt proteins (excluding Wnt5b, Wnt8b, and Wnt9b) and 10 Frizzled (Fzd) receptors (excluding Fzd4 and Fzd5) was found to be elevated in TECs (Gross, 2021). Additionally, gene overexpression of active β-catenin proteins in RTCs induces epithelial dedifferentiation and EMT, further promoting fibrosis (Figure 1) (Hu et al., 2022).

### 2.10 Notch

The Notch pathway is a highly conserved signaling network essential for fibrosis development in various organs and tissues. Under pathological conditions, Notch1 is a key mediator of RF, with HIF-1α closely related to Notch1. The effects of Notch activation vary depending on the cellular and tissue environment, as well as the physiological or pathological state. Notch signaling interacts extensively with other pathways. RF is characterized by increased collagen and ECM deposition, myofibroblast proliferation, leukocyte migration, epithelial cell dysfunction, and capillary thinning (Gao et al., 2021). Both Notch1 and Notch2 are expressed during kidney development, and the kidney exhibits high levels of Notch1 and Notch2 expression (Mukherjee et al., 2019). However, unlike in the lung, Notch activity is not present in healthy human and rodent kidneys (Niranjan et al., 2008). In contrast, Notch signaling is reactivated in various kidney disease models where upregulation of TGF-β and Notch pathways induces RTEC dedifferentiation (Hirata et al., 2021). This reactivation stimulates EMT transcription, ultimately leading to RF (Figure 1).

### 2.11 Hedgehog genes

The hedgehog signaling pathway is a significant communicator and key player in the pathogenesis of RF (Fania et al., 2020). Hedgehog signaling is an evolutionarily conserved pathway crucial for regulating mammalian embryonic development (Robbins et al., 2022). First identified in a genetic screen of *Drosophila* in 1980, the vertebrate hedgehog protein was discovered in 1993 (Krauss et al., 1993). In mammals, there are three hedgehog ligands: Sonic hedgehog (Shh), Indian hedgehog (Ihh), and Desert hedgehog (Dhh), with Shh being the most extensively characterized (Lum and Beachy, 2004). Increasing evidence indicates that Shh signaling is activated in RF, suggesting a potential link between dysregulated Shh signaling and the development of RF (Rauhauser et al., 2015) (Figure 1).

Shh signaling plays a significant role in the inducibility and localization of CKD. Typically, the hedgehog factor is not expressed in healthy RTECs. However, during RF, Shh signaling is reactivated, contributing to the disease’s progression (Zhou et al., 2014).

Shh signaling is primarily produced by damaged tubules and serves as a key communicator between damaged TECs and mesenchymal fibroblasts. This interaction leads to the activation and proliferation of fibroblasts, which play a central role in renal fibrogenesis (He et al., 2024). Meanwhile, hedgehog signaling primarily targets mesenchymal cells, and Wnt and Notch signaling specifically target TECs, indirectly promoting RF through partial mesenchymal transformation. Shh signaling also mediates changes in ECM components during renal fibrogenesis.

## 3 Drug therapy

Several novel antifibrotic drugs are being developed based on the mechanisms leading to RF. For example, pirfenidone is a non-toxic small-molecule drug that inhibits ECM accumulation without affecting normal matrix turnover; it has demonstrated efficacy in kidney injury models (Song et al., 2022). Relaxin, a hormone in the insulin-like growth factor (IGF) family, also exhibits antifibrotic properties (Wang et al., 2022b). BMP-7, as previously discussed, effectively reduces glomerular and interstitial area and prevents glomerulosclerosis, showing superior results compared to enalapril. Recent studies suggest that targeting ferroptosis may also inhibit RF and help mitigate renal lesions.

## 4 Intervention study of Chinese medicine on RF

### 4.1 Overview of TCM for RF

RF is not explicitly categorized in TCM. Instead, it is often described based on clinical manifestations such as “deficiency,” “lumbago,” “edema,” “stranguria,” “urinary turbidity,” “retention of urine,” “dysuria,” and “frequent vomiting.” These manifestations are associated with various TCM categories, including “deficiency,” “intermingled dampness-turbidity and stasis,” “blood stasis,” and “internal toxicity” (Yang et al., 2019). The pathogenesis of RF in TCM aligns closely with these categories. Pathological changes in RF are similar to those observed in the TCM concepts of “deficiency” and “toxicity.” Specifically, the mechanisms of RF can be understood as involving “blood stasis,” “turbid toxin,” and “blockage of the kidney,” leading to reduced blood supply and renal failure. Blood stasis, which exacerbates kidney damage, is considered a significant mechanism in “toxic damage to kidney channels.” Therefore, “eliminating blood stasis and clearing kidney channels” is crucial for slowing the progression of CKD. Prof. Dai Enlai’s theory suggests that “if blood stasis is not eliminated, it is difficult to restore kidney qi.” He proposes that CKD is characterized by “poison damaging kidney collaterals” with the capillary mass of the glomerulus being analogous to the “kidney collaterals” in TCM. Additionally, he highlights the role of the immune response, stating that the activation and release of coagulation factors, fibrinolysis, kinin, and inflammatory factors lead to endothelial damage of renal blood vessels, promoting RF (Enlai, 2014). Blood stasis, resulting from impaired coagulation and abnormal blood rheology due to platelet aggregation after endothelial damage, further damages the renal complex and impairs renal function.

### 4.2 TCM treatment for RF

Clinical treatment of RF is based on the therapeutic principle of invigorating the spleen and kidney, clearing heat and promoting diuresis, promoting blood circulation for removing blood stasis, resolving turbidity, and eliminating toxins. The selection of TCM is based on tonifying medicines, clearing away heat, dispelling dampness and diarrhea, and activating blood circulation. Modern medicine believes that the mechanism of Chinese medicine to improve RF may be related to tonifying the kidney, activating blood, clearing and detoxifying the turbidity by reducing oxidative stress, inflammatory response, regulating immunity, etc., and then improving the degree of RF to slow the progression of CKD. According to Prof. Dai Enlai, the spleen–kidney deficiency pattern is the key to the formation of RF. Treatment should first focus on tonifying the kidney, cultivating the kidney energy, and regulating the kidney qi to achieve the goal of treating the disease at its root. Common medicines used are eucommia, unprocessed rehmannia root, wolfberry fruit, etc. The treatment process advocates a slow attack to consolidate kidney qi (Enlai, 2014). Some scholars believe that heat-dampness evidence is a common type of kidney disease. Throughout the entire course of kidney disease, the occurrence, development, regression, and prognosis of heat-dampness play an extremely important role and are the main reason for kidney disease lingering. In patients who are difficult to heal and who experience recurring episodes, some experts suggest that ‘“heat-dampness” has not been eliminated; the protein is difficult to eliminate.’ It is said that when “Dampness and heat do not go, the kidney qi is difficult to recover.” Therefore, the treatment of dampness and heat is an important method of TCM treatment of kidney diseases (Dai et al., 2021). It is also believed that the pathogenesis of RF is closely related to the lungs, spleen, and kidneys. An imbalance of yin and yang between the spleen and kidneys is most prominent. The treatment process is based on warming the kidneys and clearing heat-dampness, and the emphasis is on strengthening vital qi to eliminate pathogenic factors. The medication is based on astragalus to tonify and invigorate the spleen, supplemented by unprocessed rehmannia root to nourish yin, nourish the blood, and tonify the kidneys, and cogon grass rhizome and *Atractylodes macrocephala* to clear heat and dry dampness, strengthen the spleen, and cool the blood, and identify and treat the overall medication. Some scholars believe that the deficiency of internal organs and qi is the root cause of this disease. People in middle age, whose internal organs are bad, whose lungs, spleens, and kidneys have a deficiency, who cannot transport fluid and are powerless to transform the development of the disease face deficiency, moisture, silt, and toxic interactions to form fibrosis (Yuyang et al., 2019).

#### 4.2.1 Single components of natural products used for RF treatment

We have found that monomers of herbal medicines (especially flavonoids, anthraquinones, phenols, glycosides, alkaloids, and terpenoids) can exert anti-inflammatory and antifibrotic effects via various related signaling pathways. TCM can protect the kidney by regulating TGF-β/Smad, NF-κB, MAKP, Wnt/β-catenin, PI3K/Akt/mTOR, JAK2/STAT3, Hedgehog, Notch, and other signaling pathways to reverse RF (Lin et al., 2022; Li et al., 2021b; Wang et al., 2022c). For example, pectolinarigenin (PEC), a flavonoid from *Cirsium japonicum DC*, was used in mice after surgery to reduce renal injury and tubulointerstitial fibrosis. In HEK293 cells, PEC attenuated the activity of TGF-β/Smads by inhibiting STAT3. In NRK-49 F fibroblasts, PEC blocked Smad3 and STAT3 phosphorylation while downregulating the expression of fibrosis-related genes and proteins, such as α-SMA, collagen type 1 (Col-1), and fibronectin (FN) (Sun et al., 2016).

Quercetin is a natural compound widely found in Chinese herbs such as jujube and acacia. Researchers investigated the effect of quercetin on renal injury in mice with UUO. The results showed that quercetin inhibited NF-κB signaling, regulated M1/M2 macrophage polarization, and improved the progression of RF (Lu et al., 2018). Dihydroartemisinin, an active component of *Artemisia annua*, exhibits antitumor, antibacterial, and antifibrotic properties, significantly inhibiting renal fibroblast proliferation and the phosphatidylinositol 3-kinase (PI3K) signaling pathway in mice with UUO (Chen et al., 2018b). Hirudin inhibits inflammation and reduces activation of the TGF-β pathway, thereby inhibiting EMT and ameliorating the progression of RF (Liu et al., 2022b). Resveratrol, a polyphenol with anticancer, anti-inflammatory, and antioxidant properties, can regulate the expression of Hsp70 in the kidneys of uremic rats, as well as inhibit the expression of NF-κB, thus exerting a renoprotective effect (Feng et al., 2020). Resveratrol can also inhibit renal EMT by suppressing the Smad2/3 signaling pathway in rats with UUO (Wei et al., 2020).

Li et al. (2022b) discovered that tectorigenin (TG), a compound extracted from *Belamcanda chinensis*, significantly improves tubular epithelial cell (TEC) necrosis, tubular obstruction, and interstitial inflammatory cell infiltration, as well as myofibroblast deposition and Smad3 activation, as observed through hematoxylin and eosin (HE) staining. TG enhances RF and kidney injury via the Smad3-mediated ferroptosis pathway. Shi et al. (2023) found that astragaloside IV inhibits inflammation, oxidative stress, fibrosis, endoplasmic reticulum stress, apoptosis, and ferroptosis in a mouse model of acute kidney injury (AKI) induced by cisplatin (CP), thereby alleviating renal damage. Yuan et al. (2020) reported that isoliquiritigenin (ISL) improves renal dysfunction and mitigates tubular injury in a UUO model by inhibiting inflammation and fibrosis.

Natural bioactive drugs inhibit ferroptosis-mediated RF by regulating oxidative stress. Chen et al. (2022) found that bilobalide can alleviate oxidative stress and ferroptosis by inhibiting the ubiquitination of GPX4, thereby protecting the kidneys from the effects of ferritin deposition and oxidative damage in DN. Zhu et al. (2023) reported that fomosertib improves RF lesions by inhibiting the ferroptosis pathway. Yang et al. (2022a) demonstrated that the injection of salicylate in senescence-accelerated mouse prone 8 (SAMP8) mice mitigates the effects of fibronectin (FN) and provides protective effects against age-related renal dysfunction through the inhibition of ferroptosis.

Qiu et al. (2024) found that in a cisplatin (CP)-induced AKI mouse model, intervention with gastrodin (GAS) significantly increased the expression of silent mating type information regulation 2 homolog-1 (SIRT1), indicating that GAS alleviates lipid peroxidation and inhibits ferroptosis in CP-induced AKI mice via the SIRT1/FOXO3A/GPX4 signaling pathway. Zhang et al. (2023) investigated vitexin, a flavonoid monomer extracted from medicinal plants known for its anti-inflammatory and anticancer properties. Silencing GPX4 using shRNA abolished the protective effects of vitexin against high glucose-induced HK-2 cell injury and reversed the ferroptotic effects induced by vitexin, suggesting that vitexin protects renal function by inhibiting GPX4-mediated ferroptosis. Yue et al. (2022) found that knocking out Nrf2 diminished the protective effects of epigallocatechin gallate (EGCG), indicating that EGCG reduces gentamicin-induced nephrotoxicity by inhibiting apoptosis and ferroptosis.

Yang et al. (2024) found that salvianolic acid A (SAA), a natural compound with antioxidant and anti-inflammatory properties, significantly reduced the expression of iron-responsive element binding protein 2 (IREB2) and HIF-2αat both protein and mRNA levels. Additionally, SAA reversed arsenic-induced increases in iron and ROS while upregulating levels of glutathione (GSH) and superoxide dismutase (SOD). These findings indicate that dysregulation of the HIF-2α/DUOX1/GPX4 pathway and iron homeostasis are critical mechanisms in arsenic-induced ferroptosis and that SAA can alleviate arsenic-induced ferroptosis and kidney injury. Overall, these results suggest that natural bioactive drugs serve as ferroptosis inhibitors, demonstrating efficacy in the treatment of RF by regulating oxidative stress and suppressing ferroptosis.

#### 4.2.2 Mechanism of action of natural products for RF

Chinese herbal formulas are determined according to the above-mentioned Chinese medicine theories; there are abundant studies on the treatment of RF with Chinese herbs. The forms of intervention include original Chinese medicines, Chinese medicine extracts, and Chinese medicine compound preparations, etc. The Yang Qi method of treatment tonifies Qi and nourishes Yin, activates blood circulation, removes blood stasis, clears heat, dispels dampness, nourishes the kidneys, and removes toxins. The method improves the degree of RF by alleviating hypoxia, oxidative stress, and the inflammatory response and regulating immunity, which in turn slows the progression of CKD.

**FIGURE 2:**
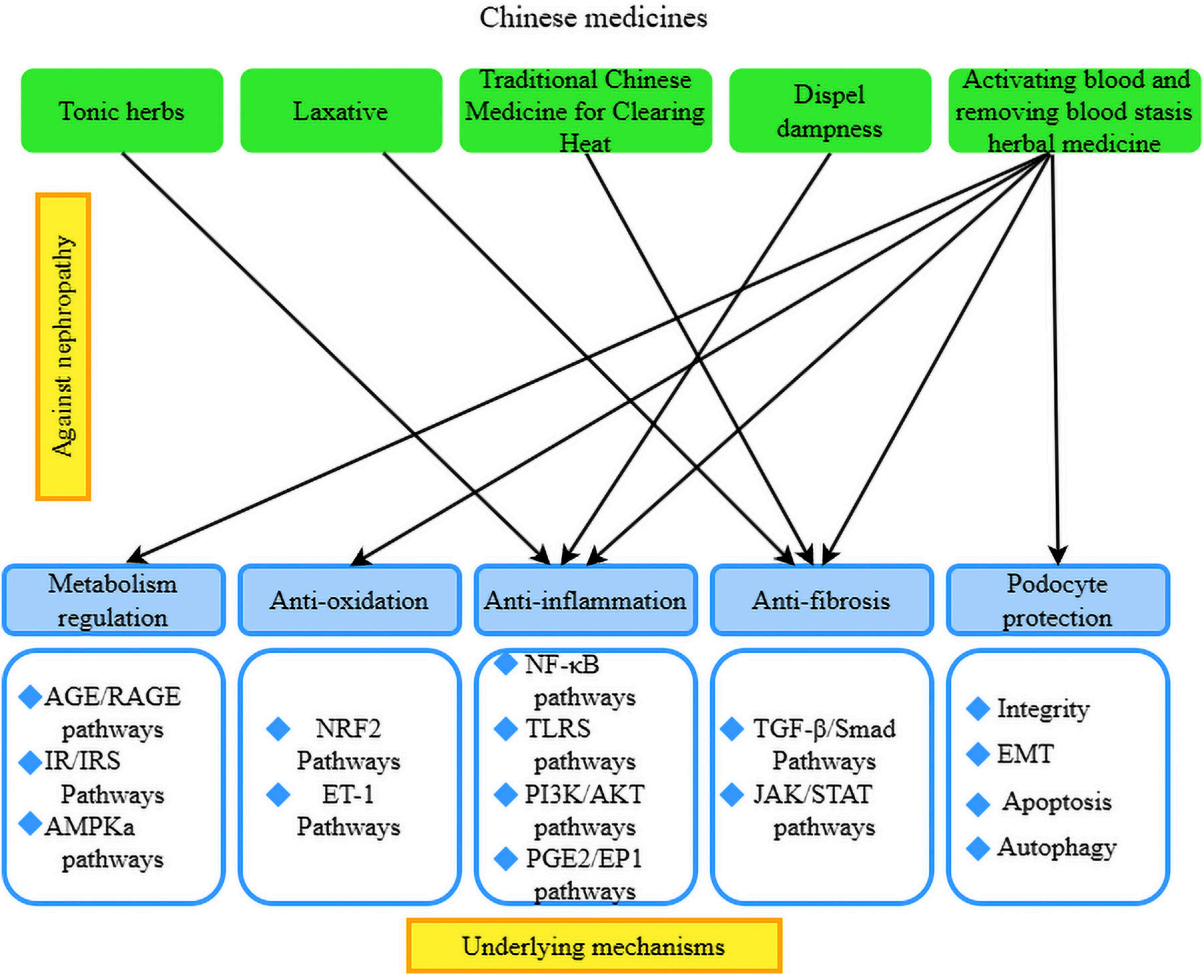
Pathogenesis of CKD prevented by TCM.

Tonic herbs: Astragalus has the effects of tonifying qi, diuresis, and muscle growth, among others. It has been widely used in the treatment of various kidney diseases. The active ingredient in astragalus, astragaloside, has various effects, such as vasodilation, prevention of endothelial dysfunction, and anti-inflammatory and antioxidant properties. Studies have shown that astragaloside reduces the deposition of ECM and the infiltration of inflammatory cells in RF and attenuates the inflammatory response (Tang et al., 2022). Additionally, it inhibits lipopolysaccharide-induced inflammatory infiltration of epithelial cells and reduces NF-кB signaling both *in vivo* and *in vitro*, thereby protecting against the progression of RF (Liu et al., 2024). Epimedium has the efficacy of tonifying kidney yang, strengthening muscles and bones, and dispelling wind-dampness. It is rich in various effective components against RF. Research has found that the total flavonoids in epimedium reduce the expression of α-SMA, TGF-β1, and Smad3 proteins in renal tissues, thereby improving RF in rats (Gai et al., 2021). Unprocessed rehmannia root induces TGF-β mRNA expression and type IV collagen accumulation, inhibits the progression of glomerulosclerosis, and prevents interstitial fibrosis and monocyte infiltration (Figure 2).

TGF-β/Smad signaling pathway: Several cellular pathways, including TGF-β/Smad, Wnt/β-catenin, and integrin/ILK, have been identified as major contributors to the generation of ECM, with TGF-β recognized as the most important profibrogenic cytokine in RF (Tang et al., 2022). A key role of TGF-β is its biological effects, which are mediated through the Smad protein signaling pathways. Therefore, inhibiting the TGF-β/Smad signaling pathway is beneficial for preventing tubulointerstitial fibrosis (TIF) and preserving renal function. Notably, evidence demonstrates that You-gui pill (YGP) has a renoprotective effect in ameliorating renal tubulointerstitial fibrosis, potentially through the suppression of TGF-β and its downstream regulatory signaling pathway, including Smad2/3 (Wang et al., 2015). Tangshen formula significantly inhibited RF, which was associated with Smad ubiquitination regulator 2 (SMURF-2)-dependent ubiquitin degradation of Smad7 and inactivation of the TGF-β1/Smad3 signaling pathway, followed by the inhibition of fibronectin and collagen I/IV expression (Zhao et al., 2016). Another study confirmed the renoprotective effect of berberine in STZ-induced mice by inhibiting the TGF-β/Smad/EMT signaling pathway (Liu et al., 2023). Similarly, Chai Huang Yi Shen granules significantly inhibited RF, 24-h proteinuria, the glomerulosclerosis index, and the tubulointerstitial fibrosis index and upregulated the expression of collagen I/IV and fibronectin through inhibition of TGF-β1/Smad3 signaling (evidenced by the upregulation of Smad7 and the downregulation of TGF-β1, TGF-β1 receptor, and Smad3). In addition to inhibiting the TGF-β1/Smad3 signaling pathway by reducing the phosphorylation of Smad2/3, oral administration of *Cortex eucommiae* also inhibited the expression of TGF-β and CTGF (key mediators of RF) in Wistar rats with STZ-induced DN, thereby decreasing the levels of blood urea nitrogen (BUN) and serum creatinine (SCr) and ameliorating RF (Niu et al., 2016).

Karunasagara et al. (2020) constructed a model of hyperglycemia-induced DN and concluded that red ginseng treatment modulated fibrotic and inflammatory mediators, such as TGF-β1, renal injury molecule-1, and advanced glycation end-products, and accelerated autophagic processes to restore renal function. Lim et al. (2019) demonstrated that Korean red ginseng (KRG) restored the expression of E-cadherin, α-SMA, and TGF-β1 in the treatment of RF. The results of a DN model showed that KRG treatment modulated fibrosis and inflammatory mediators such as TGF-β1, kidney injury molecule-1, and advanced glycation end-products, and accelerated autophagy to restore renal function. *Scutellaria baicalensis* has the efficacy of clearing heat, drying dampness, purging fire, detoxifying, stopping bleeding, and tranquilizing the fetus. *Scutellaria baicalensis* and its extracts have been found to have anti-RF effects. Baicalin exerts antifibrotic effects by inhibiting the TGF-β1-induced proliferation of rat renal interstitial fibroblasts (NRK-49F), ECM deposition, collagen synthesis, TGF-β1 expression, and Smad3 phosphorylation. Its therapeutic efficacy is reported to be better than that of baicalin alone (Wang et al., 2022c). Studies have also found that heat-clearing drugs, such as sophora flavescens, Geniposide, Shikonin, artesunate, *Centella asiatica*, cassia seed, and Ixeris sonchifolia Hance injection, can target the TGF-β1/Smad signaling pathway to prevent RF. In summary, most herbal medicines inhibit RF through the TGF-β1/Smad signaling pathway (Figure 2).

Activating blood and removing blood stasis herbal medicine: Herbs that activate blood and remove blood stasis work by promoting blood circulation, tonifying essence, and nourishing yin (Liu et al., 2022b). These herbs have been shown to attenuate mercuric chloride-induced RIF in rats by resisting oxidative stress and modulating the NF-κB signaling pathway (Wang et al., 2022d).

Dampness-expelling herbs: *Poria cocos* and *Curcuma zedoaria* are commonly used clinically as water-inducing and swelling-reducing agents. Studies have found that their extracts are effective for diuresis and kidney protection (Yang et al., 2022b). Lei Gong Teng is an expectorant for rheumatism with anti-inflammatory, antitumor, and immunosuppressive effects that is widely used in the treatment of autoimmune diseases, allergic diseases, and kidney diseases. Among its components, Lei Gong Teng polyglucoside has been shown to alleviate glomerulosclerosis in rats with adriamycin nephropathy by regulating the expression of TGF-β1, Smad3, p-Smad2/3, and Smad7 (Wan et al., 2014) (Figure 2).

Laxative herbs: Rhubarb has effects including laxative action, tapping accumulation, clearing heat and fire, cooling the blood, detoxifying toxins, expelling blood stasis, and promoting menstruation. Both rhubarb extract and its isolated compounds have demonstrated significant inhibitory effects on RF (Wang et al., 2022e).

Herbal compound formulas: Herbal compound formulas are based on TCM theories, with extensive research on their use for treating RF. Several herbal formulas are known to address RF by inhibiting endoplasmic reticulum (ER) stress. Chai Baicalin Cheng Qi Tang consists of Chai Hu, *Scutellaria baicalensis*, rhubarb, crystallized sodium sulfate, Hou Pu, *Citrus aurantium dulcis*, Yin Chen, and *Gardenia jasminoides*. In an animal model of acute pancreatitis-induced AKI, Chai Baicalin Cheng Qi Tang was found to inhibit renal ER stress indicators (BIP, XBP1, and CHOP) and apoptotic proteins (caspase-9 and cleaved caspase-3), reduce renal pathophysiological changes and apoptosis, and restore renal function. *In vitro* experiments revealed that TNF-α and IL-6 activated ER stress in HK-2 cells. *Scutellaria baicalensis* Chengqi Tang restored ER stress indicators and caspase expression levels and reduced the number of dead HK-2 cells (Yang et al., 2020). Fuzheng Huayu formula features *Salvia miltiorrhiza* as the monarch drug, activating blood circulation and removing blood stasis; *Cordyceps sinensis*, which replenishes deficiency and benefits essence and qi; peach kernel, which assists *Salvia miltiorrhiza* in blood circulation and blood stasis removal; pine pollen, which benefits qi and moistens dryness; gibberellin, which clears heat and removes toxins; and *Schisandra chinensis*, which induces menstruation. The Fuzheng Huayu formula inhibited RF by reversing the EMT of renal tubules in HgCl2-induced RF in mice and reducing Smad2/3 phosphorylation (Wang et al., 2022d). Danggui Shaoyao San (DSS) is a traditional Chinese herbal formula historically used to tonify the earth and activate the channels. Recent clinical studies suggest that DSS may ameliorate RF by alleviating tissue hypoxia, regulating autophagy, and providing nephroprotective effects (Fan et al., 2022). Zhenwu Tang (Poria, *Atractylodes macrocephala*, *Radix paeoniae alba*, Radix et Rhizoma Polygoni Multiflori, Radix et Rhizoma Zingiberis, Ginger) maintains mitochondrial integrity and improves mitochondrial oxidative phosphorylation, reflecting the theory of “yang qi tonification and strengthening.” Zhenwu Tang limits tubular fibrosis formation by enhancing tubular bioenergy (Du et al., 2022).

Chinese medicine offers unique theories on the etiology and pathogenesis of RF and the selection of treatments and remedies. While different practitioners may have their own insights, the general treatment principles remain consistent: benefiting qi and nourishing yin, activating blood circulation and removing blood stasis, clearing heat and removing dampness, nourishing the kidneys and removing toxins, and supporting yang qi throughout the prevention and treatment of RF. TCM not only addresses the local symptoms of the disease but also aims to regulate the overall integrity of the human body and its relationship with the natural environment.

Natural products derived from TCM serve as the material basis for pharmacological effects. They offer advantages such as multiple effects, targeting various pathways, and strong activity, making them a recent focus of research and new drug development.

## 5 Conclusion

RF is an extremely complex and dynamic process that involves nearly all cell types within the kidney, as well as infiltrating immune cells. Recent advances have yielded new insights into several established mechanisms underlying RF, including inflammation, hypoxia, myofibroblast activation, pericyte involvement, EMT, and the Notch and Sonic hedgehog (Shh) signaling pathways. Targeting key pathogenic mediators and signaling pathways to halt RF has demonstrated effectiveness, and some of these strategies may become clinically relevant for patients with CKD in the future.

The development of RF is asymptomatic, progressive, and irreversible. Due to its complex and poorly understood etiology, there are currently no effective preventive or therapeutic measures to delay RF. TCM offers various approaches to treating RF, emphasizing holistic concepts, evidence-based treatments, and personalized care. In recent years, TCM has made significant progress in the clinical management of RF, demonstrating particularly notable effects in anti-fibrotic therapies and other treatment modalities.

Basic research on integrating TCM and Western medicine in the treatment of CKD is still in its early stages. Enhancing the interconnection between TCM theory and modern medical practices, along with the modernization of TCM concepts, will facilitate the global adoption of TCM. This integration could provide new strategies for the clinical prevention and treatment of RF, potentially delaying the progression of CKD. Moreover, it establishes a theoretical foundation for the widespread application of Chinese medicine worldwide. This approach holds promise as a novel strategy for the clinical management of RF and could represent a significant direction in the prevention and treatment of fibrotic diseases.

Although numerous basic studies on the prevention and treatment of RF using TCM provide an objective basis for selecting clinical drugs, the material basis for their specific efficacy remains unclear. Current research faces several challenges: 1) Lack of Clinical trials: Research on TCM or specific active ingredients is often confined to cellular and animal experiments, lacking robust support from clinical trials. Discrepancies between clinical trials and animal studies, along with various intrinsic and extrinsic environmental factors, can influence results. 2) Small sample sizes: Clinical studies frequently have small sample sizes and lack multicenter, multilevel, large-scale, randomized, double-blind trials to substantiate the efficacy of TCM in treating RF. Larger studies are necessary to ensure the efficacy, tolerability, safety, and target engagement of TCM. 3) Incomplete mechanistic Understanding: Given the multi-target and multi-pathway nature of TCM, the antifibrotic mechanisms of most herbs are not fully elucidated. 4)Variability of herbal formulations: The variability in herbal formulations limits the development and standardization of TCM. More research is needed to explore the binding targets of TCM or specific active ingredients.

There is still a long way to go in understanding the pharmacological effects of Chinese medicines and their active ingredients. Additionally, identifying quality markers (Q-Markers) for TCM is crucial for accurately determining the therapeutic targets for treating RF (Lu et al., 2022). This will provide a more scientific foundation for developing future drugs aimed at RF treatment.
